# Prevalence and Characteristics of Patients with Cystic Fibrosis-Related Diabetes in Croatia

**DOI:** 10.3390/life15050815

**Published:** 2025-05-20

**Authors:** Lora Stanka Kirigin Biloš, Maja Baretić, Andrea Vukić Dugac, Krešimir Schoenwald, Ivan Bambir, Duška Tješić Drinković, Nevena Krnić, Velimir Altabas

**Affiliations:** 1 Department of Endocrinology, Diabetes and Metabolic Diseases “Mladen Sekso”, University Hospital Centre “Sestre Milosrdnice”, 10000 Zagreb, Croatia; lora.kirigin@kbcsm.hr; 2 Department of Endocrinology and Diabetology, University Hospital Centre Zagreb, 10000 Zagreb, Croatia; 3 School of Medicine, University of Zagreb, 10000 Zagreb, Croatia; 4 Cystic Fibrosis Centre for Paediatrics and Adults, University Hospital Centre Zagreb, 10000 Zagreb, Croatia; 5 Clinic for Respiratory Diseases “Jordanovac”, University Hospital Centre Zagreb, 10000 Zagreb, Croatia; 6 General Hospital Dubrovnik, 20000 Dubrovnik, Croatia; 7 Department of Paediatrics, University Hospital Centre Zagreb, 10000 Zagreb, Croatia

**Keywords:** cystic fibrosis, cystic fibrosis-related diabetes, prevalence, insulin requirements

## Abstract

This study investigated the prevalence of cystic fibrosis-related diabetes (CFRD) in the Croatian cystic fibrosis (CF) population, the age at diagnosis, insulin requirements, and the relationship between age at diagnosis and other clinical parameters. Medical records from 152 patients with genetically and laboratory-confirmed CF were reviewed through to 2025. The American Diabetes Association criteria were used to diagnose CFRD. Anthropometric and clinical data were collected from the latest medical records. A total of 17 out of 152 patients had CFRD, with a prevalence of 4.8% in the paediatric population (4/84) and 19.1% in adults (13/68). The median age of CFRD diagnosis was 14 years (range 9–22 years, SD = 3.95). Thirteen patients used insulin: one used bolus only, seven used basal-bolus multiple daily injections, and five used insulin pumps. The average total daily insulin (TDI) per kilogram (kg) body weight was 0.447 U/kg (SD = 0.429). The age at CFRD diagnosis was positively correlated with the body mass index (BMI) (*p* = 0.029). Patients requiring insulin by age 15 had higher TDI and were more likely to have CF liver disease (*p* = 0.027, *p* = 0.037, respectively). The prevalence of CFRD and age at diagnosis aligned with previous studies. Patients diagnosed at a younger age and requiring insulin earlier had lower BMIs, likely due to a faster decline in beta cell function and earlier onset of insulinopenia.

## 1. Introduction

Cystic fibrosis (CF) is the most prevalent autosomal recessive genetic condition in patients of European descent and is caused by mutations in the cystic fibrosis transmembrane conductance regulator (CFTR) protein [1]. CFTR dysfunction impairs the transport of chloride and bicarbonate in epithelial cells, leading to thick and viscous secretions in multiple organs including the lungs, pancreas, intestine, and liver [2]. Progressive respiratory impairment is the leading cause of morbidity and early mortality in patients with CF [3]. In the past few decades, advances in CF treatment—especially the introduction of CFTR modulators that partially restore CFTR function—have significantly increased the life expectancy of patients with CF. With this increased life expectancy, important non-pulmonary complications have emerged including cystic fibrosis-related diabetes (CFRD) [4].

The pathophysiology of CFRD is complex and not completely understood. CFRD shares some characteristics with both type 1 and type 2 diabetes but is a unique form of diabetes [5]. The primary cause is insulin deficiency, which is due to the dysfunction and destruction of pancreatic beta cells. However, unlike type 1 diabetes, there is no underlying autoimmune process. Instead, CFTR dysfunction causes thick, sticky secretions to accumulate in the pancreatic ducts, leading to exocrine pancreatic damage, inflammation, and fibrosis. This, in turn, damages the neighbouring pancreatic islets. In patients with CF, beta cells may also be directly affected by CFTR dysfunction. Since CFTR is expressed in beta cells, its dysfunction can impair exocytosis and glucose-stimulated insulin secretion. Thus, patients with CF exhibit a delay in first-phase insulin secretion, likely due to direct CFTR effects, and as ductal fibrosis progresses, a decrease in total insulin secretion. Like type 2 diabetes, there is also a degree of insulin resistance, driven by systemic inflammation, which periodically worsens during acute infections [6]. Hepatic insulin resistance is also seen in both CFRD and type 2 diabetes, and patients with CFRD have been shown to have an increased hepatic glucose production and a reduced suppression of hepatic glucose output by insulin [7]. Insulin resistance is further aggravated by glucocorticoids, which are sometimes used in acute lung exacerbations [8]. CFTR dysfunction may also make beta cells more susceptible to oxidative stress, resulting in impaired insulin secretion and beta cell apoptosis. Additional contributing factors include genetic predisposition related to type 2 diabetes and abnormalities in the incretin axis. Some research has suggested that patients with CFRD exhibit lower levels of glucose-dependent insulinotropic peptide (GIP) and glucagon-like peptide-1 (GLP-1) than those without diabetes and control groups [9], which may contribute to the postprandial hyperglycaemic excursions often seen in early CFRD [6].

Patients who develop CFRD have worse clinical outcomes including pulmonary and nutritional decline, increased pulmonary exacerbations, and increased mortality [8,10]. Nutritional decline is likely due to insulin deficiency-induced catabolism [11], while the increased susceptibility to infections can be explained by hyperglycaemia. Patients with CFRD have increased glucose levels on their airway surfaces, which can promote bacterial overgrowth and less effective inflammatory responses [12,13]. Thus, because CFRD is associated with worse clinical outcomes and its insidious onset, the American Diabetes Association (ADA) recommends annual CFRD screening with an oral glucose tolerance test (OGTT) in all patients with CF aged ≥10 years [14]. Screening is also recommended because timely treatment with insulin, currently the only approved therapy, improves respiratory function, body mass index (BMI), and survival for patients with CF [15,16,17].

Screening and early intervention have started to narrow the survival gap between patients with CFRD and those without it, but the risk factors of CFRD and optimal treatment strategies need more investigation [4]. Although the prevalence of CFRD has been reported in various European countries [18], most epidemiological studies were conducted before the widespread use of CFTR modulators, which may affect the onset of CFRD. Furthermore, the prevalence of CFRD in the Croatian CF population has not been reported. Thus, the primary aim of this study was to determine the prevalence of CFRD in the Croatian CF population, the age at CFRD diagnosis, insulin requirements to manage CFRD (total daily insulin/kg), and the insulin regimen used (basal only, prandial only, basal-bolus multiple daily injections, insulin pump therapy). Secondary aims were to explore the relationships between the age at CFRD diagnosis, the age of insulin introduction, and other clinical parameters.

## 2. Patients and Methods

This retrospective study was conducted at the Croatian State Referral Centre for Cystic Fibrosis in Children and Adults, which is accredited by the Ministry of Health and located at the University Hospital Centre Zagreb. This centre is the country’s primary treatment facility for CF, which cares for 98% of the Croatian CF population. Patients at this centre are routinely seen at quarterly intervals and are managed by a multidisciplinary team including a pulmonologist, gastroenterologist, endocrinologist/diabetologist, and nutritionist.

The study was conducted in accordance with the Declaration of Helsinki, and the protocol was approved by the Ethics Committee of the University Hospital Centre Zagreb (8.1-23/247-4) on 29 February 2024. All patients provided written informed consent for the entry of their data into the European CF registry, which was also used in this study.

Medical data from 152 patients with genetically and laboratory-confirmed CF were reviewed from 2019 to 2025. CFRD was diagnosed using the American Diabetes Association (ADA) criteria. CFRD was defined as fasting plasma glucose ≥ 7.0 mmol/L and/or 2 h glucose  ≥ 11.1 mmol/L, and/or glycated haemoglobin (HbA1c) ≥ 6.5% [14]. Patients who fulfilled the ADA criteria for CFRD were classified as cases regardless of their insulin requirements at their last hospital visit. Patients with type 1 diabetes were excluded (positive autoantibodies).

Clinical and anthropometric measurements were recorded from the most recent medical records. Anthropometric measurements included weight, height, and BMI. Clinical data included age, sex, CFTR genotype, insulin requirements (total basal insulin, total bolus insulin, total daily insulin, and total daily insulin/kg) and regimen used (basal only, prandial insulin only, multiple daily injections, insulin pump), type and duration of CFTR modulator therapy, presence of pancreatic insufficiency (defined as use of pancreatic enzymes), presence of CF liver disease (CFLD), forced expiratory volume in 1 s (FEV1), forced vital capacity (FVC), and whether they were lung transplant recipients. These categories were used to assess whether they were a risk factor for CFRD. Data on insulin doses (both basal and bolus) were collected from the latest diabetologist’s report. For individuals using an insulin pump, the exact basal and bolus dosage data were extracted from the most recent 30-day report. For those on basal-bolus multiple daily injections, bolus dose estimates were based on anamnestic data from the most recent diabetologist visit including insulin-to-carb ratios and basal requirements. Patients who underwent lung transplants were excluded from the FEV1 analysis. The most recent glycated haemoglobin (HbA1c) levels were recorded.

The clinical characteristics of patients with CFRD, prevalence of CFRD, age of CFRD diagnosis, and insulin requirements were assessed using descriptive statistics. The Spearman’s rank correlation coefficient was used to assess the relationships between age at CFRD diagnosis, age of insulin initiation, BMI, and HbA1c. The Mann–Whitney U test was used to assess the differences in insulin doses between patients who started insulin before and after age 15 as well as differences in the HbA1c cut-offs and insulin doses. The Fisher’s exact test was used to assess the correlation between age of insulin introduction and the age of CFRD diagnosis—before and after 15 years—and the presence of CFLD. Values of *p* < 0.05 were considered statistically significant. Medcalc (version 23.1.6) was used for statistical analysis.

## 3. Results

### 3.1. Prevalence of CFRD Within the CF Population and Age at Diagnosis

Medical records from 152 patients (68 adult, 84 paediatric) with CF were reviewed. A total of 17 out of 152 patients (11.2%) were identified with CFRD. Of the 17 patients with CFRD, the majority (76%) were ≥ 18 years. The prevalence of CFRD was 4.8% (4/84, 95% CI 1.313–11.746) in the paediatric CF population and 19.1% (13/68; 95% CI 10.591–30.470) in the adult CF population. The median age of CFRD diagnosis was 14.4 years (range 9–22 years, SD = 3.95, 95% CI 12.369–16.431), with the majority diagnosed between 9 and 15 years. More than half of the patients (53%, 95% CI 27.812–77.017) were diagnosed with CFRD by age 14, and 71% (95% CI 44.042–89.686) had their diagnosis by age 15 (Table 1).

### 3.2. Characteristics of Patients with CFRD

The characteristics of patients with CFRD are presented in Table 2 (continuous variables) and Table 3 (categorical variables). The median BMI of patients with CFRD was 23.0 kg/m^2^ (range 13.5–30.5 kg/m^2^, SD = 3.73), and the median age was 24 years (range 14–38 years, SD 6.64 years). Six patients (35.3%) had CFLD. All patients were using pancreatic enzymes for their pancreatic insufficiency, and four patients (23.5%) were using enteral nutrition. One patient was a transplant recipient and being treated with immunosuppressive therapy and corticosteroids. The majority of patients were treated with CFTR modulator therapy with an average of 36.6 months (SD = 4.27). At the last visit, only one patient was not receiving CFTR modulator therapy (transplant recipient). Only one patient was using CFTR modulator therapy at the time of CFRD diagnosis. The average FEV1 and FVC were 71.9% (SD = 19.8) and 85.4% (SD = 16.8), respectively. The average HbA1c was 6.45% (SD = 1.09). Thirteen out of seventeen patients (76.5%; 95% CI 50.101–93.189) had well-controlled diabetes (HbA1c < 7%). In terms of diabetic microvascular complications, only nine patients had a retinal screening appointment at least once, and no patients had evidence of retinopathy. As for albuminuria, only four patients were screened, two of whom had A2 albuminuria.

### 3.3. Insulin Regimens Used to Manage CFRD

The majority of patients with CFRD (76.4%; 13/17) were treated with insulin, while the remainder were managed conservatively with nutrition therapy and physical activity. No patients used non-insulin diabetes medications. Interestingly, 4 out of 17 patients were not using insulin at their last visit, despite previously requiring basal-bolus insulin therapy to manage their CFRD. In terms of the insulin regimen employed, one patient was using bolus insulin only, 41.2% (7/14 patients) were using basal-bolus multiple daily injections, and 29.4% (5/13) were using insulin pumps. The average total daily insulin/kilogram of body weight (TDI/kg) to treat diabetes was 0.854 U/kg (SD = 0.398), while the average TDI/kg in children was 0.817 (SD = 0.550) and 0.514 (SD = 0.347) in adults. When analysing the TDI/kg in all patients, including those not on insulin (assigned a value of ‘0’), the average TDI/kg was 0.447/kg (SD = 0.429).

### 3.4. Associations Between Various Clinical Parameters in Patients with CFRD

The association between various clinical parameters in patients with CFRD are shown in Table 4. The age at CFRD diagnosis and age at insulin introduction were positively correlated with BMI (Spearman’s rank correlation: *p* = 0.029, *p* = 0.025, respectively). The total daily insulin, total daily insulin/kg, and total bolus doses were positively correlated with HbA1c (Spearman’s rank correlation: *p* = 0.027, *p* = 0.033, *p* = 0.029, respectively).

When comparing groups based on age at insulin introduction—before and after age 15—using the Mann–Whitney U test, those who required insulin before age 15 needed a higher TDI/kg (*p* = 0.027), higher total daily insulin (*p* = 0.027), and higher basal insulin (*p* = 0.006). However, bolus dose requirements were not statistically significant between the groups (*p* = 0.119). The Fisher’s exact test was used to assess the correlation between age of insulin introduction—before and after 15 years—and the presence of CFLD (the outcomes were 0 or 1 for the presence of CFLD), and an association was found (*p* = 0.037), with those having insulin introduced before age 15, who were more likely to have CFLD. Age at CFRD diagnosis and presence of CFLD were not statistically different between the two groups (*p* = 0.075).

When comparing groups based on an HbA1c cut-off of 6%, using the Mann–Whitney U test, there was an association between TDI, TDI/kg, and total bolus insulin (*p* = 0.022, *p* = 0.036, *p* = 0.013, respectively), with those having an HbA1c >6% requiring higher insulin doses.

## 4. Discussion

This is the first study to report on the prevalence of CFRD in the Croatian CF population, with rates of 4.8% in the paediatric population and 19.1% in adults as well as an average age of CFRD diagnosis of 14 years. Furthermore, it provides valuable insights into the various insulin regimens used to manage CFRD and their associations with HbA1c. The study also explored the associations between the age at CFRD diagnosis, the age of insulin introduction, and other clinical parameters. The age at CFRD diagnosis and age at insulin introduction were positively correlated with the final BMI. Furthermore, patients who required insulin by age 15 had higher insulin requirements including TDI, TDI/kg, and total bolus doses. The average TDI/kg to treat diabetes was 0.447 U/kg, and patients who required insulin by age 15 were more likely to have CFLD. Insulin requirements (TDI, TDI/kg, and total bolus insulin) were positively correlated with HbA1c.

### 4.1. CFRD Prevalence

The prevalence of CFRD in the adult CF population was slightly lower than that previously reported by Moran et al., which found that 2% of children, 19% of adolescents, and 40–50% of adults were affected [19]. In their study, the prevalence of diabetes rose steadily with age, and after the age of 40 years, 45–50% were affected. In a large European Cystic Fibrosis Foundation registry study, the prevalence of CFRD in 2015 was 0.8% in patients ≤10 years, 9.7% in those aged 10–19 years, 24.1% in those aged 20–29 years, and 32.7% in those ≥30 years [18]. Thus, the prevalence of CFRD in our population is consistent with the European Registry study, albeit slightly lower.

The lower prevalence of CFRD in our adult population may be attributed to the relatively younger demographic, as most patients at our centre are under 30, and CFRD prevalence is known to increase with age [19]. Additionally, prior epidemiological studies reported on the prevalence of CFRD before the widespread use of CFTR modulators, which may delay the onset of disease. Although our study did not assess the incidence of CFRD over time, registry studies from the United States and the United Kingdom reported lower CFRD rates in the 4–5 years following ivacaftor treatment [20].

The lower CFRD prevalence in our study could also be attributed to the use of different screening methods. Not all patients were diagnosed using the gold-standard OGTT. Some patients were diagnosed using extended glucose profiles during hospital visits, and others were diagnosed using HbA1c cut-offs for diabetes, which have a lower sensitivity compared with the OGTT. Variations in CFRD prevalence across studies may also be due to inconsistent screening rates. In a 5-year prospective study, the prevalence of CFRD at a single centre increased from 11% to 24% with annual screening [21]. Furthermore, registry studies show that CF centres with lower screening rates have faster rates of pulmonary decline prior to CFRD diagnosis [22]. Despite these findings, the screening rates remain low in many CF centres, and many barriers to testing exist [4,23].

Low screening rates may be due to competing medical priorities and the perceived burden of an OGTT, which requires overnight fasting and multiple venous blood draws. Additionally, poor gastric tolerability may further reduce test adherence [24]. Thus, some centres may choose to screen with HbA1c and continuous glucose monitoring (CGM) because it is perceived as more convenient [25]. Potential strategies to increase screening uptake may include educating both patients and providers about the importance of screening, implementing systematic reminders, and establishing processes for scheduling laboratory appointments [26].

### 4.2. Age of CFRD Onset

Our study supports the current recommendations for annual CFRD screening starting at age 10, as the median age at diagnosis was 14 years. We found that more than half of the patients (53%) were diagnosed with CFRD by age 14, and 71% had their diagnosis by age 15. In a European registry study of 3553 patients, 445 (13%) were diagnosed with CFRD, and the average age of CFRD diagnosis was 13 years [22], which aligns with our study. The European Cystic Fibrosis Society (ECFS) registry does not report the date at diagnosis of CFRD, but rather the age at CFRD diagnosis is calculated as the difference between the year in which the patient began insulin and the year of birth. As many patients have abnormal OGTTs before commencing insulin, the ECFS study may have overestimated the date of CFRD diagnosis. Furthermore, the ECFS registry defines cases of CFRD as those requiring insulin, and as seen in our study, not all patients may be using insulin but still fulfil the ADA criteria for CFRD. Thus, the ECFS registry may not capture all CFRD patients, and future studies should include all CFRD patients including those not on insulin.

### 4.3. CFRD Management

Insulin is currently the only recommended therapy for CFRD; therefore, the majority of our patients (78%) were treated with insulin at their final visit. Insulin treatment was individualised based on glycaemic patterns and nutritional needs. When CGM became available, it was used to individualise treatment. Some patients with only postprandial glycaemic excursions were managed with bolus insulin alone, while those with fasting hyperglycaemia required basal insulin. Typically, the average TDI/kg in CFRD patients is in the range of 0.5–0.8 U/kg [27]. This aligns with our study, where the average TDI/kg to treat diabetes was 0.447 U/kg when considering all patients (including those assigned a value of ‘0’ at their last visit), and 0.817 U/kg when analysing only those using insulin at their final visit (13/17 patients).

Multidisciplinary care and tailored interventions are needed to optimise the outcomes for patients with CFRD [28]. Best practices for managing CFRD include individualised insulin treatment, adjustments during acute illness, and care from a diabetes specialist experienced in diabetes technology and CFRD [14].

### 4.4. CFRD Remission

Interestingly, four patients (24%) who previously required insulin had de-escalation in dosing, eventually leading to the discontinuation of insulin altogether, and were considered to have clinical remission of CFRD. At their final visit, two out of these four patients had HbA1c values consistent with normal glucose tolerance, while two patients had values consistent with prediabetes.

Clinical remission of CFRD could be explained by the introduction of CFTR modulators at our centre in 2021, which likely improved pulmonary exacerbations and the hyperglycaemia associated with acute disease. There may also be a direct effect of CFTR dysfunction on beta cells [29,30], which may improve with CFTR modulators. This was reported in a retrospective study by Gaines et al., where one-third of patients experienced remission or near remission of their diabetes following CFTR modulator therapy [31]. Similarly, Cohen et al. reported that CFTR modulator therapy improved glucose metabolism, as assessed by OGTT, and some patients experienced CFRD remission, transitioning to normal or indeterminate glucose tolerance [32]. Further research is needed to determine the optimal management of patients with apparent clinical remission of CFRD, and whether these patients remain at risk of recurrence.

### 4.5. Association Between CFRD and Clinical Parameters

As seen in our study, CFRD can progress at different rates in different people. An earlier age at CFRD diagnosis and insulin initiation was associated with a lower final BMI, and patients who required insulin by age 15 had higher insulin requirements. Patients diagnosed with CFRD at a younger age likely had a more rapid decline in beta cell function, reduced endogenous insulin secretion, and insulinopenia, leading to a catabolic environment. Additionally, the TDI, TDI/kg, and total bolus doses were statistically significantly correlated with HbA1c, suggesting that patients requiring higher doses had more advanced CFRD.

There was also an association between the age of insulin introduction and the presence of CF liver disease. CF liver disease is a known risk factor for CFRD, but the specific mechanisms underlying this association have not been thoroughly studied [33]. Elevated circulating levels of inflammatory cytokines, including interleukin IL-6, IL-1, and TNF-α, can be found in liver disease. These pro-inflammatory cytokines may alter glucose metabolism by impairing insulin signalling, promoting lipolysis, and decreasing lipogenesis and glucose oxidation [34,35]. Reduced insulin sensitivity and impaired insulin secretion have been reported in CFLD patients with portal hypertension, and these factors may contribute to the increased risk [36].

Most patients were diagnosed around the age of 14, and when patients were grouped according to the age of diagnosis and age of insulin introduction—before or after age 15—some important associations emerged including the association with CFLD and higher total insulin doses. Thus, this age group may be more vulnerable and may require more frequent disease monitoring and timely CFRD screening.

### 4.6. CFRD Chronic Complications

Screening for diabetes complications in CFRD should begin annually, starting 5 years after the diagnosis [5,14]. Unfortunately, as is the case in many CF centres including ours, screening for diabetes complications is often inconsistent due to various competing medical priorities. In a study conducted at the All Wales Adult Cystic Fibrosis Service in the UK, only 64% of patients had retinal screening appointments in 2012, and 42% had evidence of retinopathy [37]. A review of 285 CFRD patients at the University of Minnesota found that 14% with fasting hyperglycaemia and a diabetes duration of 10 years or more had microalbuminuria, and 16% had retinopathy [38]. These findings highlight the importance of retinal and albuminuria screening, especially since patients with CF are now living longer, and microvascular complications will likely become more relevant in the ageing CFRD population.

### 4.7. Strengths and Limitations

Although this study presents several interesting findings, some limitations should be addressed. The main limitation was the retrospective design and the relatively small number of patients with CFRD compared with other countries with larger CF populations. Due to the retrospective design, not all patients were diagnosed and screened for CFRD using the gold standard OGTT. Additionally, although approximately 98% of CF patients in Croatia are followed at this centre, the results are not a true representation of the country’s entire CF population. Nevertheless, this sample provides a good representation of the country’s CF population. A strength of our study was the inclusion of all CFRD patients and not just those on insulin, thus capturing a wider range of CFRD patients. Furthermore, both the age of CFRD diagnosis and the age of insulin introduction were considered.

## 5. Conclusions

In conclusion, the prevalence of CFRD in Croatia, albeit slightly lower, is consistent with other studies. However, because the landscape of CFRD is evolving due to the widespread use of CFTR modulators, large prospective studies are needed to determine the effect of CFTR modulators on CFRD development and insulin requirements. Whether the recommendations for OGTT screening will remain in place in the new era of CFTR modulators will need to be addressed in future studies. Until such studies are available, CFRD screening recommendations should be followed, and patients should be managed at CF centres by multidisciplinary teams to ensure the highest standards of care.

## Figures and Tables

**Table 1:** Frequencies of age at diagnosis of cystic fibrosis-related diabetes.

Age at CFRD Diagnosis	Counts	% of Total	Cumulative %
9	2	11.8%	11.8%
10	2	11.8%	23.5%
12	1	5.9%	29.4%
13	3	17.6%	47.1%
14	1	5.9%	52.9%
15	3	17.6%	70.6%
18	3	17.6%	88.2%
21	1	5.9%	94.1%
22	1	5.9%	100.0%

CFRD—cystic fibrosis-related diabetes.

**Table 2:** Characteristics of patients with cystic fibrosis-related diabetes continuous variables (*N* = 17).

	Mean	Median	Standard Deviation	Minimum	Maximum	95% Confidence Interval
Age	24.2	24	6.64	14	38	20.786–27.614
Height (cm)	165	161	10.7	147	182	159.5–170.5
Weight (kg)	62.7	63	14.7	35.3	95.4	55.142–70.258
BMI (kg/m^2^)	22.9	23	3.73	13.5	30.5	20.982–24.818
Age at CFRD diagnosis	14.4	14	3.95	9	22	12.369–16.431
Duration of diabetes	9.82	9	6.15	2	23	6.658–12.982
Age at insulin introduction	14.6	14	4.05	9	22	12.518–16.682
Basal insulin (U)	18.6	10	13.5	0.0	44.0	11.659–25.541
Insulin bolus (U)	16.3	12	12.4	0.0	50.0	9.925–22.675
TDI	34.2	26	23.2	6.00	86.0	22.272–46.128
TDI/kg	0.447	0.327	0.429	0.00	1.38	0.226–0.668
HbA1c%	6.45	6.40	1.09	4.70	9.30	5.890–7.010
FEV1%	71.9	73.5	19.8	38	107	62.488–81.312
FVC%	85.4	88.5	16.8	52	113	76.762–94.038
Duration of modulatory therapy (months)	36.6	35.5	4.27	32	48	34.405–38.795

BMI—body mass index, CFRD—cystic fibrosis-related diabetes, TDI—total daily insulin, HbA1c—glycated haemoglobin, FEV1—forced expiratory volume in 1 s, FVC—forced vital capacity.

**Table 3:** Characteristics of patients with cystic fibrosis-related diabetes categorical variables (*N* = 17).

		F (%)	95% Confidence Interval/%
Sex	Male	6 (35.3)	14.210–61.672
	Female	11 (64.7)	38.328–85.790
Genotype	F508 del homozygous	14 (82.4)	56.568–96.201
	F508 del heterozygous	2 (11.8)	1.458–36.441
	Other	1 (5.9)	0.149–28.689
Patients treated with insulin	No	4 (23.5)	6.811–49.899
	Yes	13 (76.5)	50.101–93.189
Insulin regimen	No insulin	4 (23.5)	6.811–49.899
	Bolus only	1 (5.9)	0.149–28.689
	Basal only	0 (0.0)	0.000–19.506
	Basal-bolus multiple daily injections	7 (41.2)	18.444–67.075
	Insulin pump	5 (29.4)	10.314–55.958
Transplant recipient	No	16 (94.1)	71.311–99.851
	Yes	1 (5.9)	0.149–28.689
Pancreatic exocrine deficiency	No	0 (0.0)	0.000–19.506
	Yes	17 (100)	80.494–100.000
Cystic Fibrosis-Related Liver Disease	No	13 (76.5)	50.101–93.189
	Yes	4 (23.5)	6.811–49.899
Treatment with modulator therapy	Ivacafor, tezacaftor, elexacaftor + ivacaftor	15 (88.2)	63.559–98.542
	Ivacafor, tezacaftor, elexacaftor	1 (5.9)	0.149–28.689
	Ivacaftor	0 (0.0)	0.000–19.506
	None	1 (5.9)	0.149–28.689
Enteral nutrition	No	4 (23.5)	6.811–49.899
	Yes	13 (76.5)	50.101–93.189
Corticosteroids or immunosuppressive therapy	No	16 (94.1)	71.311–99.851
	Yes	1 (5.9)	0.149–28.689

**Table 4:** Correlation between age of cystic fibrosis-related diabetes diagnosis, age of insulin introduction, and clinical parameters.

Parameter I	Parameter II	Spearman’s Rank Correlation Coefficient	
Age of CFRD diagnosis	BMI	*p* = 0.029	
Age of insulin introduction	BMI	*p* = 0.025	
Duration of diabetes	BMI	n.s.	
TDI	HbA1c	*p* = 0.027	
TDI/kg	HbA1c	*p* = 0.033	
Basal insulin-dose	HbA1c	n.s.	
Bolus insulin-dose	HbA1c	*p* = 0.029	
**Group I**	**Group II**	**Parameter**	**Mann–Whitney U Test**
HbA1c ≤ 6%	HbA1c ≥ 6.1%	TDI	*p* = 0.022
HbA1c ≤ 6%	HbA1c ≥ 6.1%	TDI/kg	*p* = 0.036
HbA1c ≤ 6%	HbA1c ≥ 6.1%	bolus insulin-dose	*p* = 0.013
HbA1c ≤ 6%	HbA1c ≥ 6.1%	Basal insulin-dose	n.s.
Age at insulin introduction ≤15 years	Age at insulin introduction ≥15.1 years	TDI	*p* = 0.027
Age at insulin introduction ≤15 years	Age at insulin introduction ≥15.1 years	TDI/kg	*p* = 0.027
Age at insulin introduction ≤15 years	Age at insulin introduction ≥15.1 years	Basal insulin-dose	*p* = 0.006
Age at insulin introduction ≤15 years	Age at insulin introduction ≥15.1 years	Bolus insulin-dose	n.s.
**Group I**	**Group II**	**Parameter**	**Fisher’s Exact Test**
Age at insulin introduction ≤15 years	Age at insulin introduction ≥15.1 years	CFLD	*p* = 0.037
Age of CFRD diagnosis ≤15	Age of CFRD diagnosis ≥15.1	CFLD	*p* = 0.075

TDI—total daily insulin, HbA1c—glycated haemoglobin, CFRD—cystic fibrosis-related diabetes, CFLD—cystic fibrosis live disease, ns—not significant.

## Data Availability

The original contributions presented in the study are included in the article, further inquiries can be directed to the corresponding author.
